# Understanding Nurses’ Perceptions of Patient‐Centered Care: The Influence of Interprofessional Collaboration

**DOI:** 10.1155/nrp/3672766

**Published:** 2026-08-06

**Authors:** Trang Thi Thuy Ho, Binh Thi Diem Vo, Thao Thi Phuong Hoang

**Affiliations:** ^1^ Faculty of Nursing, Hue University of Medicine and Pharmacy, Hue University, Hue, Vietnam, hueuni.edu.vn

**Keywords:** interprofessional collaboration, nurse perceptions, nursing care, patient-centered care

## Abstract

**Background:**

Patient‐centered care and interprofessional collaboration are fundamental to high‐quality healthcare. This study investigated nurses’ perceptions of patient‐centered care and interprofessional collaboration and its factors.

**Methods:**

A descriptive cross‐sectional survey was conducted. A total of 320 nurses from multiple hospitals in Vietnam participated in the study. The Index of Interprofessional Team Collaboration for Expanded School Mental Health and Patient‐Centered Care Scale were used to assess interprofessional collaboration and perceptions of patient‐centered care. Data were analyzed using multiple linear regression and mediation analysis.

**Results:**

Nurses reported moderate perceptions of interprofessional collaboration (2.68 ± 0.41) and relatively high perceptions of patient‐centered care (3.83 ± 0.57). Multiple regression analysis revealed that perceptions of interprofessional collaboration (*p* < 0.001), patient‐centered care training (*p* = 0.001), work experience (*p* = 0.001), and living area (*p* = 0.001) were significant predictors of patient‐centered care perception. Interprofessional collaboration significantly mediated the relationship between patient‐centered care training and patient‐centered care perception.

**Conclusion:**

Nurses’ perceptions of interprofessional collaboration significantly influence their views on patient‐centered care, and strengthening these perceptions through targeted training and organizational support could enhance care quality and consistency.

## 1. Introduction

Patient‐centered care and interprofessional collaboration are recognized as foundational components of contemporary nursing practice. Patient‐centered care emphasizes respect for patients’ preferences, needs, and values, ensuring that patient values guide clinical decisions [1–3]. Interprofessional collaboration involves effective communication, shared decision‐making, and mutual respect among healthcare professionals from diverse disciplines, enabling the delivery of integrated and high‐quality care [4–7]. Nurses occupy a pivotal role in operationalizing both patient‐centered and collaborative practices. Their continuous presence at the bedside positions them uniquely to advocate for patients, facilitate communication across professional boundaries, and promote a culture of teamwork and respect. By facilitating collaboration and advocacy, they support the establishment of a care environment that is both holistic and seamlessly coordinated across disciplines.

The transition to patient‐centered care is gaining increasing recognition in Vietnam’s healthcare system [8, 9]. Implementing patient‐centered care has the potential to improve health outcomes, increase patient satisfaction, enhance care coordination, and promote more efficient use of health resources [9–12]. Additionally, increasing demand for quality health services highlights the urgent need to reorient the health system toward a more patient‐centered model [13, 14]. Healthcare system shifted from traditional models that primarily revolved around the clinician’s perspective to a more collaborative and personalized approach [15]. This paradigm shift requires health care providers to develop stronger interpersonal skills, adopt a more holistic understanding of patient needs, and foster trust‐based relationships. Therefore, embracing patient‐centered care not only aligns with global health reform trends but also represents a strategic response to the challenges in service delivery.

Interprofessional collaboration is regarded as a cornerstone of comprehensive, high‐quality care, facilitating coordinated and team‐based approaches among diverse health professionals to enhance safety, efficiency, and patient‐centeredness in service delivery [16, 17]. The integration of interprofessional collaboration into routine clinical practice remains at an early stage of development in the Vietnamese healthcare system [18]. Despite initial efforts to incorporate interprofessional education into curricula, implementation remains fragmented and lacks widespread adoption [19–21]. Traditionally, medical and nursing education has focused heavily on discipline‐specific training, with limited emphasis on interprofessional teamwork or shared clinical decision‐making [5]. Moreover, health care system continues to operate within a hierarchical, physician‐centered structure, which poses challenges to fostering collaborative care [18]. Nurses constitute the majority of the healthcare workforce; however, limited decision‐making authority continues to constrain their meaningful participation in interprofessional teams [22]. These structural and educational barriers highlight the need for systemic reforms to promote interprofessional collaboration and support a more integrated, team‐based model of care.

Enhancing collaboration is an effective strategy for promoting patient‐centered care [23]. In complex clinical settings, effective interprofessional collaboration enables seamless coordination and continuity of care across multiple healthcare providers [24, 25]. Effective interprofessional collaboration strengthens coordination and continuity of care and promotes an environment that enables nurses to actively advocate for patients’ needs and preferences [23, 26]. Moreover, by reducing occupational stress and improving job satisfaction, effective interprofessional collaboration supports the provision of empathetic, responsive, and individualized care, aligning with the core principles of the patient‐centered care model [27]. As healthcare systems globally shift toward value‐based care, investment in team building interventions and interprofessional collaboration is becoming increasingly vital to achieving optimal patient outcomes and organizational performance.

Although substantial international evidence has demonstrated the importance of interprofessional collaboration in promoting patient‐centered care [28, 29], empirical evidence within the Vietnamese healthcare context remains limited, particularly in primary‐level hospitals and resource‐constrained regions. Moreover, relatively little attention has been directed toward understanding how interprofessional collaboration influences nurses’ perceptions of patient‐centered care in routine clinical practice. This research gap is particularly important in Vietnam, where hierarchical healthcare structures, workforce limitations, and disparities in healthcare resources hinder collaborative practice and the effective delivery of patient‐centered care. Therefore, generating context‐specific evidence is essential to inform evidence‐based policies and develop interventions tailored to the realities of healthcare practice in Vietnam and other resource‐limited settings.

This study aimed to examine the relationship between interprofessional collaboration and nurses’ perceptions of patient‐centered care. Understanding these relationships inform the development of targeted interventions to strengthen teamwork and improve patient outcomes in clinical practice. The findings are expected to contribute to the growing body of evidence on collaborative healthcare practice and provide practical insights for healthcare administrators, educators, and policymakers seeking to enhance patient‐centered care through effective interprofessional collaboration.

## 2. Methods

### 2.1. Study Design and Locale of the Study

A cross‐sectional descriptive study design was employed to assess nurses’ perceptions of interprofessional collaboration and patient‐centered care. The study was conducted from January 2024 to December 2024 across multiple primary‐level hospitals in the central region of Vietnam. A convenience sampling strategy was used to recruit frontline nursing staff directly involved in patient care from hospitals with similar resource‐constrained and community‐based healthcare environments.

### 2.2. Sampling and Participants

Eligible participants were registered nurses currently employed in primary‐level hospitals in the central provinces of Vietnam who had at least 6 months of clinical experience in direct patient care. Nurses were excluded if they declined participation or were on leave during the data collection period.

The sample size was calculated using G^∗^ Power for multiple linear regression analysis. The calculation was based on the primary study objective of examining the association between interprofessional collaboration and patient‐centered care. Assuming a small effect size (*f* ^2^ = 0.05), a significance level of 0.05, a statistical power of 0.95, and two primary predictor variables, the minimum required sample size was estimated to be 312 participants. Although additional covariates were included in the final regression model and mediation analysis was subsequently conducted, these analyses were exploratory and secondary to the primary objective on which the sample size estimation was based.

To account for potential nonresponse and incomplete questionnaires, 320 nurses were invited to participate. After excluding 24 questionnaires because of incomplete responses or missing data, 296 participants were included in the final analysis. Although the final sample size was slightly below the calculated target, it remained adequate for the planned regression analyses based on recommended participant‐to‐predictor ratios and acceptable statistical power for the primary analyses.

### 2.3. Study Instruments

General information collected from the study participants included age, sex, ethnicity, education level, years of experience, department or specialty, and prior participation in or interest in attending courses on interprofessional collaboration and patient‐centered care.

Perceptions of interprofessional collaboration were assessed using the Index of Interprofessional Team Collaboration for Expanded School Mental Health with a 26‐item instrument developed by Mellin et al. with four subscales reflection on process, professional flexibility, newly created professional activities, and role interdependence [30]. Although originally developed in a school mental health setting, the instrument assesses key dimensions of interprofessional collaboration that are relevant across multidisciplinary healthcare environments, including hospital nursing settings, such as communication, shared decision‐making, professional role clarity, and team coordination. Each item is rated on a 5‐point Likert scale ranging from “Strongly Disagree” to “Strongly Agree.” The original instrument demonstrated strong internal consistency, with Cronbach’s alpha coefficients of 0.91, 0.91, 0.84, and 0.80 for the respective subscales [30]. The questionnaire was translated into Vietnamese using a standardized forward–backward translation process following the recommendations of Ozolins et al. [31]. The translated instrument was reviewed by bilingual experts to ensure semantic and conceptual equivalence. A pilot study involving 20 nurses further confirmed the clarity and reliability of the Vietnamese version. The Vietnamese version demonstrated good overall reliability, with a Cronbach’s alpha coefficient of 0.90. Subscale‐specific Cronbach’s alpha coefficients in the current sample were 0.85 for reflection on process, 0.88 for professional flexibility, 0.78 for newly created professional activities, and 0.77 for role interdependence, indicating acceptable to good internal consistency across domains.

Perceptions of patient‐centered care were measured using a 20‐item instrument developed by Sidani and colleagues [32], which is organized into three domains: holistic care, collaborative care, and responsive care. Each item is rated on a 5‐point Likert scale ranging from “Not at all” to “Very much so.” In the original version, the content validity indices for all three subscales were 100%, and each item within a subscale loaded onto a single factor. The reliability coefficients were 0.66 for holistic care, 0.70 for collaborative care, and 0.42 for responsive care. The instrument was subsequently translated into Vietnamese by Adetunji et al. [14], with all 20 items receiving excellent validity ratings. Permission to use the questionnaire was obtained from the original authors. In the current study, the instrument demonstrated good overall internal consistency, with a Cronbach’s alpha coefficient of 0.83. Subscale‐specific Cronbach’s alpha coefficients were 0.80 for holistic care, 0.90 for collaborative care, and 0.75 for responsive care, indicating acceptable reliability for assessing perceptions of patient‐centered care among hospital nurses in Vietnam.

### 2.4. Data Collection Procedure

Data collection began after approval from the institutional ethics review board. Before participation, written informed consent was obtained from all participants, ensuring they were fully informed about the study’s purpose, procedures, potential risks and benefits, confidentiality protections, and their right to withdraw at any time without penalty. Trained research assistants conducted the data collection in hospital settings during working hours to minimize disruption. Participants were encouraged to ask questions, and all concerns were addressed promptly. Completed questionnaires were anonymized using unique codes and securely stored in locked cabinets and password‐protected digital files accessible only to the research team. All procedures adhered to the ethical principles outlined in the Declaration of Helsinki.

### 2.5. Data Analysis

Data were analyzed using IBM SPSS Statistics version 29.0. Participant characteristics were summarized using descriptive statistics. Categorical variables were presented as frequencies and percentages, whereas continuous variables were expressed as means and standard deviations. Multiple linear regression analyses were conducted to examine factors associated with perceptions of patient‐centered care. Binary independent variables were dummy coded, with “Yes” coded as 1 and “No” coded as 0. A simple mediation analysis was conducted using the PROCESS macro for SPSS (Model 4) with bias‐corrected bootstrap confidence intervals. Indirect effects were estimated using 5,000 bootstrap samples, and statistical significance was determined when the 95% confidence interval did not include zero.

### 2.6. Ethical Consideration

Ethical approval for the study was granted by the Institutional Review Board of X University (H2024/393). Participants were informed of the voluntary nature of their involvement, their right to withdraw at any time without penalty, and the confidentiality of their responses. Written informed consent was secured from all participants before data collection commenced.

## 3. Results

### 3.1. Characteristics Profile of the Participants

Of the 320 nurses initially surveyed, data from 296 nurses were included in the final analysis after excluding incomplete or invalid questionnaires. The majority of participants were aged 30–40 years (60.5%), female (86.8%), and of the Kinh ethnic group (88.2%). Nearly half (45.9%) had 10–20 years of work experience, and most lived in rural areas (66.6%). Regarding educational attainment, 49.3% held a college degree, while 43.9% possessed a bachelor’s degree. Participants represented various departments, with the largest proportions working in Internal Medicine (20.3%), Obstetrics and Gynecology (19.3%), and other specialties (24.0%). In terms of professional development, 74.3% had attended training on interprofessional collaboration and 69.9% had received training related to patient‐centered care. Notably, most participants expressed a continued need for further training in both interprofessional collaboration and patient‐centered care. Detailed results are presented in Table 1.

**TABLE 1:** Characteristics of study population (*n* = 296).

Variable		*n* (%)	Perception of interprofessional collaboration		Perceptions of patient‐centered care	
			Mean (SD)	*p*	Mean (SD)	*p*
Age	< 30	31 (10.5)	2.78 (0.43)	0.95^a^	3.77 (0.56)	0.71^a^
	30–40	179 (60.5)	2.68 (0.42)		3.80 (0.57)	
	40–50	68 (23.0)	2.66 (0.40)		3.90 (0.54)	
	> 50	18 (6.1)	2.65 (0.51)		4.04 (0.61)	

Sex	Female	257 (86.8)	2.66 (0.42)	0.04^b^	3.83 (0.56)	0.91^b^
	Male	39 (13.2)	2.80 (0.36)		3.83 (0.60)	

Ethnicity	Kinh	261 (88.2)	2.66 (0.42)	0.06^b^	3.84 (0.56)	0.39^b^
	Other	35 (11.8)	2.81 (0.36)		3.76 (0.64)	

Living area	Urban	99 (33.4)	2.73 (0.36)	0.13^b^	3.66 (0.60)	< 0.001^b^
	Rural	197 (66.6)	2.66 (0.44)		3.92 (0.53)	

Religion	Buddhism	34 (11.5)	2.74 (0.36)	0.64^a^	3.60 (0.55)	0.001^a^
	Catholicism/Protestantism	10 (3.4)	2.64 (0.31)		3.66 (0.51)	
	No religion	200 (67.6)	2.67 (0.45)		3.92 (0.55)	
	Other	52 (17.6)	2.70 (0.33)		3.69 (0.58)	

Education level	Diploma (2‐year nursing training program)	17 (5.7)	2.86 (0.39)	0.26^a^	3.93 (0.73)	0.59^a^
	College degree (3‐year nursing training program)	146 (49.3)	2.69 (0.41)		3.79 (0.58)	
	Bachelor’s degree (4‐year nursing training program)	130 (43.9)	2.64 (0.42)		3.87 (0.53)	
	Postgraduate degree	3 (1.0)	2.85 (0.29)		3.75 (0.47)	

Work experience (years)	< 10	131 (44.3)	2.72 (0.42)	0.47^a^	3.71 (0.62)	0.001^a^
	10–20	136 (45.9)	2.64 (0.40)		3.91 (0.49)	
	Over 20	29 (9.8)	2.70 (0.47)		4.05 (0.55)	

Department currently working in	Internal medicine	60 (20.3)	2.82 (0.31)	0.06^a^	3.75 (0.50)	< 0.001^a^
	Surgery	39 (13.2)	2.59 (0.44)		3.76 (0.39)	
	Pediatrics	15 (5.1)	2.67 (0.32)		4.04 (0.42)	
	Intensive Care and Toxicology	54 (18.2)	2.61 (0.40)		3.74 (0.56)	
	Obstetrics and Gynecology	57 (19.3)	2.60 (0.40)		3.65 (0.63)	
	Other specialties (ENT/Infection Control/Dentistry)	71 (24.0)	2.73 (0.49)		4.11 (0.58)	

Attended interprofessional collaboration training	Yes	220 (74.3)	2.74 (0.40)	< 0.001^b^	3.80 (0.56)	0.07^b^
	No	76 (25.7)	2.50 (0.41)		3.93 (0.56)	

Attended patient‐centered care training	Yes	207 (69.9)	2.75 (0.39)	< 0.001^b^	3.76 (0.57)	0.001^b^
	No	89 (30.1)	2.52 (0.42)		4.00 (0.52)	

Need to attend a course on interprofessional collaboration	Yes	266 (89.9)	2.70 (0.41)	0.03^b^	3.84 (0.56)	0.88^b^
	No	30 (10.1)	2.53 (0.42)		3.80 (0.62)	

Need to attend a course on patient‐centered care	Yes	276 (93.2)	2.69 (0.41)	0.02^b^	3.83 (0.57)	0.30^b^
	No	20 (6.8)	2.49 (0.41)		3.95 (0.58)	

^a^Kruskal–Wallis test.

^b^Mann–Whitney *U* test.

Statistically significant differences in interprofessional collaboration scores were observed based on sex, participation in interprofessional collaboration and patient‐centered care training, and the perceived need for such training. Male nurses reported higher collaboration scores than their female counterparts (*p* = 0.04). Nurses who had attended interprofessional collaboration training scored significantly higher than those who had not (*p* < 0.001). Similarly, nurses who had received patient‐centered care training reported higher collaboration scores than those without such training (*p* < 0.001). Additionally, those who expressed a need for further collaboration training had significantly higher scores than those who did not (*p* < 0.005).

Significant differences in perceptions of patient‐centered care were observed across various demographic and professional characteristics. Nurses working in rural areas reported higher perception scores than those in urban settings (*p* < 0.001). Religious affiliation was also associated with differences in perception (*p* = 0.001), with nurses reporting no religion showing the highest scores (3.92 ± 0.55) and those identifying as Buddhist the lowest (3.60 ± 0.55). Departmental differences were significant (*p* < 0.001), with the highest scores observed among nurses in specialties such as ENT, Infection Control, or Dentistry (4.11 ± 0.58), followed by Pediatrics (4.04 ± 0.42). In terms of experience, nurses with over 20 years of work reported the highest scores (4.05 ± 0.55), compared to those with less than 10 years (3.71 ± 0.62, *p* = 0.001). The bivariate analysis showed that nurses who had not attended patient‐centered care training reported significantly higher unadjusted patient‐centered care perception scores than those who had attended training (*p* = 0.001).

### 3.2. Nurses’ Perception of Interprofessional Collaboration and Patient‐Centered Care

The mean overall score for interprofessional team collaboration among nurses was 2.68 ± 0.41. Among the subscales, reflection on process achieved the highest mean score (3.75 ± 0.59), followed by newly created professional activities (2.81 ± 0.55), role interdependence (2.73 ± 0.48), and collective ownership of goals (2.67 ± 0.78). Professional flexibility recorded the lowest mean score (2.48 ± 0.52). Regarding perceptions of patient‐centered care, the overall mean score was 3.83 ± 0.57. Among its components, provision of information/instructions had the highest mean score (3.98 ± 0.66), followed by collaborative care (3.92 ± 0.66), responsive care (3.84 ± 0.63), and attendance to patients’ needs (3.49 ± 0.66) (Table 2).

**TABLE 2:** Descriptive statistics of interprofessional collaboration and patient‐centered care among nurses (*n* = 296).

Variable	Mean ± SD	Min–max
Interprofessional team collaboration	2.68 ± 0.41	1.62–3.77
Reflection on process	3.75 ± 0.59	1.88–5.00
Role interdependence	2.73 ± 0.48	1.33–4.00
Newly created professional activities	2.81 ± 0.55	1.00–4.00
Professional flexibility	2.48 ± 0.52	1.00–4.00
Collective ownership of goals	2.67 ± 0.78	1.00–4.00
Perceptions of patient‐centered care	3.83 ± 0.57	2.00–4.90
Holistic care	3.74 ± 0.59	1.98–5.00
Collaborative care	3.92 ± 0.66	1.29–5.00
Responsive care	3.84 ± 0.63	2.00–5.00

### 3.3. Analysis of Factors of Perceptions of Patient‐Centered Care Among the Nurses

A multiple linear regression analysis was conducted to identify factors associated with perceptions of patient‐centered care among nurses. The overall regression model was statistically significant, *F* (4, 291) = 13.85, *p* < 0.001, explaining 16.0% of the variance in perceptions of patient‐centered care (*R* ^2^ = 0.160; adjusted *R* ^2^ = 0.148). Perceived interprofessional collaboration emerged as a significant predictor (*p* < 0.001), indicating that higher levels of collaboration were associated with more positive perceptions of patient‐centered care. Attendance at training on patient‐centered care was also significantly associated with the outcome (*p* = 0.001), as was work experience (*p* = 0.001). Additionally, living area significantly predicted perceptions of patient‐centered care (*p* = 0.001) (Table 3).

**TABLE 3:** Multiple linear regression analysis predicting perceptions of patient‐centered care (*n* = 296).

Variable	Unstandardized coefficient		Standardized coefficient	*t*	*p*	
	*β*	Std. error	*β*			
Perceptions of interprofessional collaboration	0.33	0.08	0.24	4.34	< 0.001	*R* ^2^ = 0.160, adjusted *R* ^2^ = 0.148, *F* = 13.85, *p* < 0.001
Attended training on patient‐centered care	0.24	0.07	0.20	3.48	0.001	
Work experience	0.17	0.05	0.19	3.50	0.001	
Living area	0.23	0.07	0.19	3.52	0.001	

**FIGURE 1:**
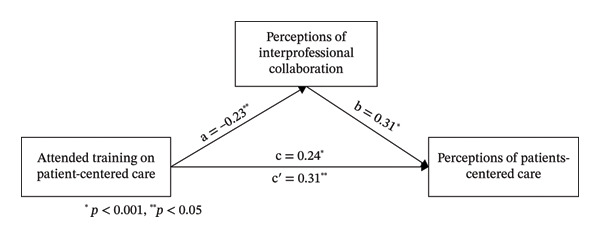
Bootstrap mediating effect analysis of perceptions of patient‐centered care among nurses.

Table 4 presents the results of the multiple regression and mediation analyses, highlighting both direct and indirect effects on perceptions of patient‐centered care. Attendance at patient‐centered care training was a significant negative predictor of perceptions of interprofessional collaboration (*p* < 0.001), but exerted a significant positive direct effect on perceptions of patient‐centered care (*p* < 0.001). Additionally, perceived interprofessional collaboration positively predicted perceptions of patient‐centered care (*p* < 0.001). A significant indirect effect was also observed: training attendance influenced perceptions of patient‐centered care through its impact on interprofessional collaboration. This mediation pathway was statistically substantial (*β* = −0.07, SE = 0.03, *p* < 0.05, 95% CI [−0.1248, −0.0288]) (Figure 1).

**TABLE 4:** Results of the mediation analysis (*n* = 296).

Path	*B*	SE	*t*	*p*	95% CI (LLCI, ULCI)	*β*
Path a: Attended training on patient‐centered care ⟶ Perceptions of interprofessional collaboration	−0.23	0.05	−4.43	< 0.001	(−0.3256, −0.1254)	−0.55
Path b: Perceptions of interprofessional collaboration ⟶ Perceptions of patient‐centered care	0.31	0.08	3.95	0.001	(0.1562, 0.4663)	0.23
Path c (total effect): Attended training on patient‐centered care ⟶ Perceptions of patient‐centered care	0.24	0.07	3.40	0.008	(0.1011, 0.3783)	0.42
Path c′ (direct effect): Attended training on patient‐centered care ⟶ Perceptions of patient‐centered care (controlling for perceptions of interprofessional collaboration)	0.31	0.07	4.37	< 0.001	(0.1702, 0.4496)	0.55
Indirect effect (a × b)	−0.07	0.03	—	—	(−0.1248, −0.0294)	−0.12

## 4. Discussion

The results indicate that participants generally perceived their care as patient‐centered, with the highest ratings observed in collaborative care, followed by responsive care and holistic care. Compared to the findings reported by Adetunji and colleagues, the current study reflects slightly lower perceptions of patient‐centered care overall, suggesting potential contextual or cultural differences in how care is delivered and experienced [14]. Further comparison with a study validating the patient‐centered care scale among Korean nurses similarly demonstrated that collaborative care was rated highest, followed by holistic care, with responsive care receiving the lowest score [33]. These findings suggest that nurses perceive themselves as actively engaging in multidimensional patient‐centered practices, particularly in the domain of collaborative care. The prominence of collaborative care reflects the increasing integration of team‐based approaches within hospital systems, where communication and shared decision‐making are emphasized as core competencies. The relatively strong perception of collaborative practices indicates alignment between institutional policies and frontline nursing experiences. It suggests that nurses recognize the value of coordinated teamwork in enhancing patient engagement, safety, and continuity of care, and supports the theoretical robustness of patient‐centered care framework, particularly in emphasizing the central role of interprofessional collaboration. Additionally, while nurses conceptually endorse addressing patients’ needs, practical constraints, including staffing shortages, time limitations, and high patient acuity, hinder full implementation. Holistic care requires deeper interpersonal engagement and individualized assessment, which could be challenging in resource‐constrained settings. The findings therefore highlight a potential gap between professional ideals and operational realities.

Our findings demonstrate a moderate overall level of interprofessional team collaboration, with variability across specific dimensions; however, these scores were comparatively lower than those reported by Mellin and colleagues [30], who observed substantially higher levels of role interdependence, professional flexibility, and newly created professional activities. This reflects transitional stages in team development, where interprofessional collaboration is recognized conceptually but not yet deeply embedded in routine workflows. Among the subdomains, reflection on process demonstrated the highest mean score, suggesting that healthcare professionals actively engage in self‐assessment and team‐level reflection, an essential component of the Interprofessional Education Collaborative core competency of Teams and Teamwork [34], which emphasizes shared learning, mutual support, and continuous performance improvement. This indicates that nurses perceive active engagement in evaluating and improving team processes. Limited professional flexibility could indicate rigid role boundaries or insufficient empowerment to engage beyond discipline‐specific responsibilities. Similarly, moderate ratings in collective ownership of goals suggest that while team members work alongside one another, a fully integrated sense of shared mission and mutual accountability might not be consistently achieved. The results emphasize the importance of targeted interprofessional education initiatives aimed at enhancing role clarity, adaptability, shared goal setting, and cross‐disciplinary innovation.

Several factors were significantly associated with these perceptions of patient‐centered care and interprofessional collaboration among primary nurses. Notably, male nurses reported higher scores for interprofessional collaboration compared to their female counterparts, suggesting potential sex‐related differences in communication styles, leadership engagement, or confidence within team‐based environments. This aligns with findings by Wrześniewska Wal et al., who reported that female nurses were more likely to express a need for interprofessional collaboration [35]. However, this finding contrasts with previous research, which reported that sex did not significantly influence interprofessional collaboration among primary healthcare professionals [36]. This discrepancy reflects contextual differences in healthcare systems, professional cultures, or the evolving role of sex in team dynamics across different countries.

Our findings indicate that living area, religion, work experience, and current department significantly influenced nurses’ perceptions of patient‐centered care. Religious affiliation influenced perceptions; clinical nurses without a religious affiliation reported higher patient‐centered care scores than those identifying as Buddhist or Christian. While the underlying mechanisms remain speculative, this finding may reflect differing value systems, belief structures, or approaches to patient engagement that influence clinical interactions. Notably, living area emerged as a significant predictor, with nurses practicing in rural settings reporting higher patient‐centered care scores than their urban counterparts. This finding may reflect closer provider–patient relationships in less urbanized environments, where continuity of care and more personalized communication are often more feasible. Within the Donabedian framework, this highlights how structural characteristics, such as practice environment and community size, could shape care processes and ultimately impact care quality [37].

In terms of professional background, nurses with more than 20 years of experience reported the highest levels of patient‐centered care perception. This aligns with Benner’s Novice to Expert Theory, which suggests that greater clinical experience fosters more intuitive, individualized, and holistic approaches to care [38]. Departmental differences were also evident. Nurses working in departments such as ENT, Infection Control, and Dentistry demonstrated the highest perceptions of patient‐centered care, followed by those in Pediatrics. These specialties inherently encourage more focused, personalized interactions due to the nature of patient needs and service delivery, reinforcing the importance of practice context in shaping care experiences. Supporting these findings, previous studies have identified age, years of experience within a specific unit, and employment in high acuity settings as significant factors influencing nurses’ perceptions of patient‐centered care [23]. Similarly, Bardhia et al. identified the practice environment, age, and educational level as key predictors of patient‐centered care [39].

The regression model predicting nurses’ perceptions of patient‐centered care was statistically significant (*F* = 13.85, *p* < 0.001), explaining 16.0% of the variance in patient‐centered care perceptions (adjusted *R* ^2^ = 0.148). The findings indicate that perceived interprofessional collaboration, participation in patient‐centered care training, work experience, and living area were significant factors associated with nurses’ perceptions of patient‐centered care. Notably, perceived interprofessional collaboration, which remains a relatively emerging concept within the Vietnamese healthcare context, demonstrated a meaningful contribution to patient‐centered care perceptions. Furthermore, the mediation analysis revealed that perceived interprofessional collaboration partially mediated the relationship between participation in patient‐centered care training and perceptions of patient‐centered care. Specifically, training participation showed a direct positive association with patient‐centered care perception, while perceived interprofessional collaboration positively influenced this relationship.

An important finding of this study was the discrepancy between the unadjusted and adjusted associations related to participation in patient‐centered care training. In the bivariate analysis, nurses who had not attended training reported higher patient‐centered care perception scores than those who had attended training. However, after controlling for demographic and professional characteristics in the multivariable regression analysis, participation in patient‐centered care training emerged as a positive predictor of patient‐centered care perception. This finding suggests that the relationship between training participation and perceptions of patient‐centered care might be influenced by contextual, demographic, and professional factors included in the adjusted model. One possible explanation is that nurses who attended training might have developed greater awareness of patient‐centered care principles and standards, leading to more critical evaluations of their own clinical practice and organizational environments, whereas nurses without formal training have based their perceptions primarily on routine clinical experiences. In addition, the negative association observed between training participation and perceived interprofessional collaboration in the mediation analysis indicates that educational interventions alone are insufficient to strengthen collaborative practice in the absence of supportive organizational structures, effective teamwork cultures, and opportunities for interdisciplinary engagement. Supporting this, a qualitative study using a descriptive phenomenological approach identified “lack of collaborative practice” as a central theme in exploring primary healthcare providers’ perceptions and experiences of interprofessional collaboration [18]. These findings highlight the complexity of translating educational interventions into clinical practice and suggest that, although training enhances knowledge and awareness related to patient‐centered care, its effectiveness depends on healthcare organizations providing environments that facilitate interprofessional communication, shared decision‐making, and collaborative care delivery. Therefore, educational strategies should be implemented alongside organizational interventions aimed at fostering supportive workplace cultures and strengthening interprofessional collaboration to achieve sustainable improvements in patient‐centered care.

This study has several limitations that warrant consideration. First, the cross‐sectional design precludes causal inferences regarding the relationships among the study variables. Second, the use of self‐reported measures has introduced response and social desirability bias. Finally, as the study was conducted within a limited geographic area and specific healthcare settings, the generalizability of the findings to other institutional or cultural contexts may be constrained.

## 5. Conclusion

This study highlights the significant influence of interprofessional collaboration, training participation, work experience, and living area on nurses’ perceptions of patient‐centered care. The partial mediation by perceived interprofessional collaboration underscores its vital role in enhancing patient‐centered care outcomes. However, the observed disconnect between training and clinical practice suggests that educational initiatives alone are insufficient. To effectively improve patient‐centered care, healthcare organizations should foster supportive environments that promote authentic interprofessional collaboration alongside comprehensive training programs. In addition, future research should employ longitudinal or intervention‐based designs to better examine causal relationships among interprofessional collaboration, professional training, and patient‐centered care.

## Funding

The authors received no financial support for the research, authorship, or publication of this article.

## Ethics Statement

Ethical approval for the study was granted by the Institutional Review Board of X University (H2024/393). Participants were informed of the voluntary nature of their involvement, their right to withdraw at any time without penalty, and the confidentiality of their responses. Written informed consent was secured from all participants before data collection commenced.

## Conflicts of Interest

The authors declare no conflicts of interest.

## Data Availability

The data that support the findings of this study are available upon request from the corresponding author. The data are not publicly available due to privacy or ethical restrictions.
